# Anxiety, depression, and quality of life in parents of children with congenital hyperinsulinism

**DOI:** 10.1007/s00431-022-04486-9

**Published:** 2022-05-04

**Authors:** Marcia Roeper, Henrike Hoermann, Roschan Salimi Dafsari, Felix Koestner, Ertan Mayatepek, Sebastian Kummer, Christina Reinauer, Thomas Meissner

**Affiliations:** Department of General Pediatrics, Neonatology and Pediatric Cardiology, Medical Faculty, University Children’s Hospital Düsseldorf, Heinrich-Heine-University, Moorenstr. 5, 40225 Düsseldorf, Germany

**Keywords:** Depression, Anxiety, Mental health, Quality of life, Family burden, Congenital hyperinsulinism, Chronic disease

## Abstract

This study aimed to assess mental health, family burden, and quality of life (PQoL) in parents of children with persistent congenital hyperinsulinism (CHI). Forty-eight individual CHI parents (75% female) completed self-reported questionnaires and screening tools for anxiety (GAD-7), depression (PHQ-8), PQoL (ULQIE), and family burden (FaBeL). Additional data on sociodemographics, social support, and child- and disease-related data were recorded. 29.8% of parents showed major depressive symptoms and 38.3% had a probable general anxiety disorder, including 20.8% who had both. The family burden was moderate and assessment of PQoL yielded average scores. Neurological impairment in an affected child (*p* = .002 and *p* < .001, respectively) and lower working hours (*p* = .001 and *p* = .012, respectively) were the strongest predictors of worse GAD-7 and PHQ-8 scores. Furthermore, lower working hours (*p* = .012) and comorbidities in the affected child (*p* = .007) were significantly associated with lower PQoL. Mothers had worse GAD-7 scores (*p* = .006) and lower PQoL (*p* = .035) than fathers. Indication of sleep disturbance was associated with worse PHQ-8 scores (*p* = .003), higher family burden (*p* = .039), and reduced PQoL (*p* = .003). A higher number of caretakers besides parents was associated with decreased family burden (*p* = .019), improved PQoL (*p* < .001), and lower scores for anxiety (*p* = .016) and depressive (*p* = .021) symptoms.

*Conclusion*: Symptoms of depression and anxiety are alarmingly prevalent in parents of children with CHI. Psychological screening of parents should be initiated to ensure early identification of psychological strains and psychosocial support should be offered as needed. A good support network and regular work activities can improve parental mental health and well-being.

**Table:** 

**What is Known:** *• Psychosocial strains and reduced quality of life are common in parents of chronically ill children.*	
**What is New:** *• In this first study evaluating mental health, family burden, and quality of life in parents of children with congenital hyperinsulinism (CHI), symptoms of depression and anxiety were alarmingly prevalent.* *• Parents of children with CHI should receive regular psychological screening and psychosocial support should be offered as needed. A good support network and regular work activities can improve parental mental health and well-being.*

**What is Known:**

*• Psychosocial strains and reduced quality of life are common in parents of chronically ill children.*

**What is New:**

*• In this first study evaluating mental health, family burden, and quality of life in parents of children with congenital hyperinsulinism (CHI), symptoms of depression and anxiety were alarmingly prevalent.*

*• Parents of children with CHI should receive regular psychological screening and psychosocial support should be offered as needed. A good support network and regular work activities can improve parental mental health and well-being.*

## Introduction

Congenital hyperinsulinism (CHI) is a rare disorder but the major cause of persistent hypoglycemia in children [1]. Dysregulated insulin secretion from pancreatic beta-cells results in recurrent and often severe hypoglycemia, which poses a significant risk for hypoglycemic brain injury [2]. Neurological sequelae affect up to 50% of patients [3–7]. Parents of affected children are therefore often in fear of severe hypoglycemia and the resulting complications. CHI is a heterogeneous disease in terms of genetics, natural course, treatment options, and disease severity. While some children may be stabilized with oral diazoxide, others require more intense pharmacological treatment and dietary management with frequent carbohydrate intake and sometimes even tube feeding [8]. In children with focal disease or if medical treatment has failed, subtotal pancreatectomy may be performed. However, surgical treatment often leads to comorbidities such as insulin-dependent diabetes or pancreatic exocrine dysfunction [6, 9].

For many families, disease management is time-consuming, emotionally challenging, and demands a great amount of personal commitment. Given the rarity of the disease, the caregivers are often the only “experts” around.

Previous reports have shown that psychosocial strains and reduced parental quality of life (PQoL) are common in parents of chronically ill children [10–14], and especially in CHI [8]. However, the burden of parenting a child with CHI has not yet been systematically evaluated. In this study, we aimed to assess the prevalence of depression and anxiety symptoms, family burden, and PQoL of parents caring for a child with CHI to identify predictors of adverse psychosocial outcomes that can be addressed by offering early counseling and adequate psychosocial support.

## Materials and methods

In a cross-sectional study, anxiety, depression, family burden, and PQoL in parents of children with persistent CHI were assessed in an anonymous online survey using SoSciSurvey (Leiner. 2019. Munich, Germany). Parents were eligible to participate in the study if they were proficient in German and if their child had been diagnosed with persistent CHI, i.e., if the disease had been present for at least 6 months. Eligible parents were recruited during clinic appointments at the University Children’s Hospital Duesseldorf, by email, telephone, or letter. Additionally, the survey link was distributed via the newsletter of the German CHI support group “Kongenitaler Hyperinsulinismus e.V.” One hundred families were contacted and both parents were invited to participate in the study. Data collection began in June 2019 and was completed in March 2020. All individuals gave informed consent before completing the questionnaires.

### Measurements

Sociodemographic data such as parental gender, age, marital status, educational level, and current employment status were surveyed. Furthermore, information on social support and CHI disease-related data, such as frequency of blood glucose measurements, hypoglycemic episodes, use of continuous glucose monitoring (CGM), neurodevelopmental outcome, and comorbidities, were collected and self-reported in the questionnaire. Four standardized self-report instruments were used to assess parents’ psychosocial strains:

#### Anxiety

The Generalized Anxiety Disorder Scale-7 (GAD-7) is a brief 7-item self-report questionnaire to evaluate symptoms of anxiety over the previous 2 weeks. Items are rated on a 4-point scale from 0 (“not at all”) to 3 (“nearly every day”), providing a total sum score of 0–21 points to describe the severity of anxiety symptoms. Cut-off scores for mild, moderate, and severe anxiety were 5, 10, and 15 points, respectively [15]. The cut-off ≥ 10 points is used to determine a probable general anxiety disorder, as it was associated with high sensitivity (89%) and specificity (82%) in the validation study [15]. In the current study, Cronbach’s alpha was 0.88.

#### Depression

Depressive symptoms were assessed using the Patient Health Questionnaire (PHQ‐8) [16]. It is a screening instrument for self-assessment of depressive symptoms in the past 2 weeks and consists of eight criteria for the diagnosis of depressive disorders according to the Diagnostic and Statistical Manual of Mental Disorders, Fourth Edition (DSM-IV). The ninth DSM-IV criterion on suicidal thoughts or actions is omitted in the PHQ-8. Items are scored on a 4-point scale from 0 (“not at all”) to 3 (“nearly every day”) and a total sum score of 0–24 points is calculated to describe disease severity. Current depression is defined by a total score ≥ 5 with four categories of severity: mild 5–9, moderate 10–14, moderately severe 15–19, and severe depression 20–24. The cut-off ≥ 10 points is used to define a probable major depressive disorder, as it yielded high sensitivity (≥ 99%) and specificity (92%) in the validation study [17]. Cronbach’s alpha was calculated at 0.88.

#### Parental quality of life

The Ulm Quality of Life Inventory for Parents of Chronically Ill Children (ULQIE) was used to assess PQoL [18]. It is a 29-item self-report questionnaire specifically developed for parents of children with chronic illness. It consists of a total score and five subscales depicting the dimensions physical and daily functioning, satisfaction with family, emotional stability, self-development, and well-being. Answers are given regarding the past 7 days on a 5-point scale ranging from 0 (“never”) to 4 (“always”). Higher scores indicate higher PQoL. Cronbach’s alpha ranged from 0.66 to 0.87 for the subscales and 0.94 for the total score.

#### Family burden

The Family Burden Questionnaire (FaBeL), the German version of the Impact on Family Scale [19], was used to assess the burden of the child’s chronic condition on the family. The self-report questionnaire consists of 33 items on five subscales: daily/social burden, personal strains, financial burden, impact on siblings, and problems in coping. Furthermore, a total score excluding the six sibling-related items is computed. Answers are given on a 4-point Likert scale ranging from 1 (“strongly agree”) to 4 (“strongly disagree”) with higher scores indicating higher burden. In this study, only the total score (Cronbach’s alpha = 0.85) was used for comparative analysis.

### Statistical analysis

Data were analyzed using SPSS Statistics version 25.0 (IBM Inc., Armonk, NY, USA). Standard descriptive statistics were computed to assess baseline data. Cronbach’s alpha was calculated for all scales to test for internal consistency, with a value > 0.7 considered acceptable. Normality of distribution was determined via Kolmogorov–Smirnov-test. For univariate analysis, Student’s *t*-test, Mann–Whitney-*U* test, Pearson’s chi-squared test, Fisher’s exact test, Spearman’s correlations, and univariate regression were calculated when applicable.

All significant variables for the total item scores from univariate analysis were entered into multivariate regression models with stepwise backward elimination to assess the impact of possible predictors on psychosocial outcome and PQoL.

Continuous variables are presented as mean with standard deviation (SD) or range for parametric variable and as median with interquartile range (IQR) for non-parametric data. Categorical variables are reported as number (*n*) and percent (%). A *p*-value < 0.05 was considered statistically significant.

The study was approved by the institutional review board of the Medical Faculty of the Heinrich-Heine-University Duesseldorf, Germany (2019–420-ProspDEvA), and was performed following the Declaration of Helsinki.

## Results

In total, 48 parents of children with CHI participated in the study, 36 (75%) mothers and 12 (25%) fathers. The mean parental age was 41.5 years (range 26–54). 85.5% (*n* = 41) were married or in a stable relationship and 14.5% (*n* = 7) were divorced, single, or widowed. On average, respondents had 2 (IQR 1) children. Mean weekly working hours were 25.5 (SD 10.5) for mothers and 43.9 (SD 10.9) for fathers (*p* < .001). Besides themselves, parents had a median of 2 (IQR 2) additional caretakers for their child. In total, 33% of parents (15 mothers, 1 father, *p* = .033) indicated that they currently or previously received psychological care. There were no further gender-specific differences in sociodemographic status or child and disease-specific data (Tables 1 and 2).

**Table 1:** Characteristics and associations of study participants

**Variable**	**Value**	***p*-value**			
		*GAD-7*	*PHQ-8*	*ULQIE total score*	*FaBeL total score*
**Age** *(years; mean, range)*^b^	41.5 (26–54)	.395	.791	.164	.873
**Female gender** *(n, %)*^a^	36 (75)	**.006**^**c**^	.102	**.035**^**c**^	.843
**Number of children** *(median, IQR)*^b^	2 (1)	.175	.192	.219	.678
**Relationship status** *(n, %)*^a^					
Married/in stable relationship	41 (85.5)	.072	.571	.77	.555
**Education** *(n, %)*^a^					
Secondary education or higher	29 (60.4)	.177	.303	.86	.51
**Employment status** *(n, %)*					
Respondent currently employed^a^	39 (81.3)	.971	.418	.306	.317
Partner currently employed^a^ (*n* = 43)	35 (81.4)	.97	.855	.794	.274
Both parents employed^a^	28 (58.3)	.575	.492	.394	.293
Working full-time^a^ (*n* = 34)	18 (52.9)	.061	.06	.163	.69
Respondents’ weekly working hour (*n* = 39) *(median, IQR)*^b^	23.5 (20)	**.01**^**d**^	**.004**^**d**^	**.013**^**e**^	.557
Partners’ weekly working hours (*n* = 35) *(median, IQR)*^b^	40 (2)	.426	.940	.379	.734
**Current or prior psychological care** *(n, %)*^a^	16 (33.3)	**.031**^**c**^	**.003**^**c**^	.05	.959
**Respondent has chronic disease** *(n, %)*^a^	2 (4.2)	.456	.845	.191	.898
**Sleep disturbance during last 7 days** *(n, %)*^a^	21 (43.8)	.203	**.007**^**c**^	**.003**^**c**^	**.039**^**c**^
**Number of independent caretakers for the affected child besides parents** *(median, IQR)*^b^	2 (2)	**.016**^**d**^	**.021**^**d**^	** < .001**^**e**^	**.019**^**d**^

*n* number or total number, *%* percent, *IQR* interquartile range. Total number was 48 unless stated otherwise. *GAD-7* Generalized Anxiety Disorder Scale-7, *PHQ-8* Patient Health Questionnaire-8, *ULQIE* Ulm Quality of Life Inventory for Parents of Chronically Ill Children, *FaBeL* Family Burden Questionnaire

^a^Student’s *t*-test

^b^Univariate regression or Spearman’s correlation depending on normality of distribution. ^c^Worse scores

^d^Negative correlation

^e^Positive correlation

**Table 2:** Characteristics of children with CHI and disease-specific data (n = 48)

**Variable**	**Value**	***p*-value**			
		*GAD-7*	*PHQ-8*	*ULQIE total score*	*FaBeL total score*
Age* *(years; mean, range)*^b^	8.8 (0.6–22)	.206	.915	.446	.863
**Neurodevelopmental impairment**^a^	12 (25)	**.009**^**c**^	**.024**^**c**^	**.011**^**c**^	.831
Comorbidities**^a^	17 (35.4)	.107	.083	**.009**^**c**^	.494
**Sibling with chronic disease**^a^	5 (10.4)	.175	.773	.926	.423
**Using continuous glucose monitoring (CGM)**^a^	21 (43.8)	.427	.863	.977	.547
**Daily blood glucose measurements**^a^	28 (58.3)	.211	.58	.132	.928
Prior severe hypoglycemia***^a^	26 (54.2)	.468	.248	.89	.509
**Weekly hypoglycemia < 60 mg/dL**^a^	17 (35.4)	.589	.676	.203	.897

Values are presented as number (*n*) and percent (%) if not stated otherwise

*GAD-7* Generalized Anxiety Disorder Scale-7, *PHQ-8* Patient Health Questionnaire-8, *ULQIE* Ulm Quality of Life Inventory for Parents of Chronically Ill Children, *FaBeL* Family Burden Questionnaire

^*^Children were diagnosed with CHI between 1997 and 2019; **Comorbidities included epilepsy, heart defect, hypothyroidism, diabetes type III, hearing deficit, growth hormone deficiency, kidney disease, atopic disease, scoliosis, AD(H)D, and depression.;***Prior severe hypoglycemia means with seizure or loss of consciousness

^a^Student’s *t*-test

^b^Univariate regression

^c^Worse scores

### Anxiety and depression

In total, 29.8% (*n* = 14) of parents had major depressive symptoms according to the PHQ-8 and 38.3% (*n* = 18) had a probable general anxiety disorder according to the GAD-7, including 20.8% (*n* = 10) who had both (Table 3). GAD-7 and PHQ-8 scores were positively correlated in the study (*p* < .001). Spearman’s correlation showed that worse scores on both the GAD-7 and the PHQ-8 were significantly correlated with lower PQoL in total and on all subscales (each *p* ≤ .001). The analysis showed that parents who worked more weekly hours had better GAD-7 scores (*p* = .01) and better PHQ-8 scores (*p* = .004). Having fewer caretakers for the CHI child besides parents was associated with worse PHQ-8 scores (*p* = .021) and worse GAD-7 scores (*p* = .016). Mothers had significantly worse GAD-7 scores than fathers (*p* = .006). No association was found between PHQ-8 scores and gender. Current or prior psychological care was associated with worse scores on both GAD-7 (*p* = .003) and PHQ-8 (*p* = .031). Parents of children with neurological impairment had significantly worse scores on both the GAD-7 (*p* = .009) and the PHQ-8 (*p* = .024). Parents who indicated sleep disturbance had worse PHQ-8 scores (*p* = .007).

**Table 3:** Severity of anxiety or depressive symptoms according to GAD-7 and PHQ-8 scores in the study cohort compared to the German general population

**Results**	**GAD-7**		**PHQ-8**	
	*Study cohort*	*German general population *[20]	*Study cohort*	*German general population *[21]
**Mean scores** (*SD)*	8.9 (5.2)	3.6 (3.3)	8.0 (5.6)	
** ≥ 10 points**	38.3 (18)		29.8 (14)	9.2
** < 10 points**	61.7 (29)		70.2 (33)	
**Category of severity**				
*Mild*	38.3 (18)		41.7 (20)	
*Moderate*	23.4 (11)		19.1 (9)	6.3
*Moderately severe*	-		4.3 (2)	2.9*
*Severe*	14.9 (7)		6.4 (3)	

Values are presented as percent and number (*n*) if not stated otherwise

Patient Health Questionnaire-8 (PHQ‐8) category cut-offs for depressive symptoms: none 0–4, mild 5–9, moderate 10–14, moderately severe 15–19, and severe 20–24 point. Cut-off for probable major depressive disorder ≥ 10 points

Generalized Anxiety Disorder Scale-7 (GAD-7) category cut-offs for anxiety symptoms: none 0–4, mild 5–9, moderate 10–14, and severe 15–21 points. Cut-off for probable general anxiety disorder ≥ 10 points; ^*^Moderately severe and severe cut-off are summarized

Higher total family burden (FaBel total score) and daily/social burden (FaBeL subscale 1) were correlated with worse PHQ-8 scores (*p* = .01 and .017) but not with anxiety symptoms. Furthermore, there were no significant correlations between GAD-7 or PHQ-8 scores and parental or patients’ age, marital status, number of children, or partners’ weekly working hours. Comparative analysis showed no association between a probable general anxiety disorder (GAD-7 score ≥ 10 points) or a probable major depressive disorder (PHQ-8 score ≥ 10 points) and any sociodemographic, or child- and disease-specific data.

In multivariate regression analysis with backward elimination, neurological impairment in the affected child (*p* = .002 in both models) and respondent’s weekly working hours (*p* = .001 in both models) remained significant predictors of both the GAD-7 and PHQ-8 scores and explained 33.6% and 38.4% of the variance (Table 4).

**Table 4:** Multiple regression models with stepwise backward elimination

**Variable**	**GAD-7**	**PHQ-8**	**ULQIE**
**Adjusted** ***R***^**2**^ **for model**	.336	.384	.516
**Gender**	n.s	n.i	n.s
**Psychological care**	n.s	n.s	n.s
**Weekly working hours**	*B* = −.181; *p* = .001	*B* = −.221; *p* = .001	*B* = .016; *p* = .012
**Comorbidities**	n.i	n.i	*B* = −.496; *p* = .007
**Neurodevelopmental impairment**	*B* = 5.131; *p* = .002	*B* = 5.897; *p* = .002	n.s
**Number of independent caretakers**	n.s	n.s	*B* = .183; *p* < .001
**FaBeL total score**	n.i	n.s	n.i
**Sleep disturbance during last 7 days**	n.i	n.s	n.s

*n.i.* not included, *n.s.* not significant, *GAD-7* Generalized Anxiety Disorder Scale-7, *PHQ-8* Patient Health Questionnaire-8, *ULQIE* Ulm Quality of Life Inventory for Parents of Chronically Ill Children

### PQoL

Parents reported the highest PQoL on the subscale for “satisfaction with family” and lowest for “self-development” (Table 5). Lower total PQoL was seen in mothers (*p* = .035), parents who indicated sleep disturbance (*p* = .003), parents who had undergone prior psychological intervention or were in current psychological care (*p* = .050), and parents who had only few caretakers for the affected child besides themselves (*p* < .001). Caregivers also reported lower total PQoL if their child had any comorbidities (*p* = .009) or neurodevelopmental impairment (*p* = .011). Higher working hours correlated with higher total PQoL (*p* = .013). No associations were found between PQoL and other sociodemographic or child- and disease-specific data. Separate analyses of associations with the ULQIE subscales are presented in Table 6. In multivariate regression analysis with stepwise backward elimination, the number of caretakers for the CHI child besides parents (*p* < .001), comorbidities (*p* = .007), and weekly working hours (*p* = .012) were significant predictors of the cohorts’ PQoL and explained 51.6% of the variance (Table 4).

**Table 5:** Family burden and PQoL

**Scale results**	**Mean (SD)**	**Range**	***Cronbach’s*** **alpha**
**FaBel scores**			
Daily/social impact	2.28 (0.63)	1.2–3.8	0.88
Personal strains	2.45 (0.62)	1–3.6	0.45
Financial burden	2.05 (0.69)	1–3.5	0.63
Impact on siblings	0.59 (0.51)	1–3	0.69
Problems in coping	1.87 (0.59)	1–3	0.28
FaBeL total score *(without sibling items)*	2.39 (0.46)	1.3–3.2	0.85
**ULQIE scores**			
Physical and daily functioning	2.37 (0.73)	0.85–3.85	0.87
Satisfaction with family	2.82 (0.81)	0.67–4	0.83
Emotional stability	2.15 (0.98)	0–3.75	0.81
Self-development	1.57 (0.87)	0–4	0.85
Well-being	2.48 (0.75)	1–3.75	0.66
ULQIE total score	2.33 (0.67)	0.82–3.62	0.94

Family Burden Questionnaire (FaBel): 4-point Likert rating scale ranging from 1 (“strongly agree”) to 4 (“strongly disagree”). Higher scores indicate higher family burden. Ulm Quality of Life Inventory for Parents of Chronically Ill Children (ULQIE): 5-point Likert rating scale ranging from 0 (“never”) to 4 (“always”). Higher scores indicate higher PQoL

**Table 6:** Detailed analysis of ULQIE subscales

**Variable**	***p*-value**			
	*U1*	*U2*	*U3*	*U4*
**Female gender**^a^	n.s	0.27^c^	.009^c^	n.s
**Psychological care**^a^	n.s	.016^c^	.027^c^	n.s
**Comorbidities**^a^	.036^c^	< .001^c^	n.s	n.s
**Neurodevelopmental impairment**^a^	.006^c^	.001^c^	n.s	n.s
**Weekly working hours**^b^	n.s	n.s	.005^d^	n.s
**Number of independent caretakers**^b^	< .001^d^	n.s	.006^d^	< .001^d^
**Number of children**^b^	n.s	n.s	.042^d^	n.s
**Sleep disturbance during last 7 days**^a^	.001^c^	n.s	.028^c^	.006^c^

Ulm Quality of Life Inventory for Parents of Chronically Ill Children (ULQIE) subscales: U1 — physical and daily functioning, U2 — satisfaction with family, U3 — emotional stability, U4 — self-development. The subscale U5 for well-being was excluded from the analysis because internal consistency according to Cronbach’s alpha did not reach the accepted value of > .7

^a^Student’s *t*-test

^b^Spearman’s correlation

^c^Worse scores

^d^Positive correlation

### Family burden

On average, parents reported moderate family burden. The lowest burden was recorded on the FaBeL subscale for “impact on siblings” and the highest burden on the subscale for “personal strains” (Table 5). Parents who had only few independent caretakers for the CHI child and who indicated sleep disturbance had significantly higher total family burden (*p* = .019 and *p* = .039, respectively). Other sociodemographic, disease- or child-related data had no impact on the FabeL total score, and no correlation was found between perceived family burden and PQoL.

## Discussion

In this first study assessing the psychosocial burden of parenting a child with CHI, caregivers reported pronounced rates of anxiety and depressive symptoms.

Anxiety symptoms according to the GAD-7 mean score and depressive symptoms in the PHQ-8 were significantly more prevalent in the cohort than in large samples of the German general population [20, 21] (Table 3).

Higher levels of depression and anxiety have been reported in parents of children with numerous chronic diseases [22, 23]. van Oers et al. found that practical problems in daily life and parenting stress were the strongest predictors of anxiety and depression, while illness-related data had no impact on the psychological outcome [22].

Surprisingly, the frequency of hypoglycemia had no impact on anxiety and depressive symptoms in parents of children with CHI. However, in multiple regression analysis, the strongest predictor was a child’s neurological impairment. It has been previously reported that caring for a disabled child is associated with a high caregiving burden and psychological morbidity [24–26].

Comparable to our study, mothers have been reported to have significantly more symptoms of anxiety than fathers [22] and prior or current psychotherapy was a predictor of both anxiety and depression [23]. Furthermore, sleep quality has a significant effect on PQoL and psychological outcome or vice versa [27].

In this study, self-assessment yielded average scores for PQoL. The result is comparable to other studies using the same instrument for parents of chronically ill children [18, 28, 29]. Parents in our analysis reported the lowest scores for “self-development” and highest scores for “satisfaction with family life,” as previously described [12, 18, 28, 29]. It can be assumed that parents often put their children’s needs above their own, leaving them little time for personal development due to the time-consuming disease management.

Mothers reported significantly lower PQoL than fathers. This result may indicate their role as the child’s primary caretaker, as there were significantly lower weekly working hours reported by mothers compared to fathers. Because treating a child with CHI is often demanding and challenging, primary caretakers carry the main burden of managing daily medical and social care, which impacts both their mental health and work activities.

Having a chronically ill child was associated with reduced parental employment in several studies [30, 31] and higher rates of parents working part-time compared to parents of healthy children [32].

Interestingly, a higher number of weekly working hours were associated with decreased symptoms of depression and anxiety and increased PQoL in our study. Job gratification and distraction from daily coping with the child’s illness may explain this finding. However, the parents’ ability to work is likely influenced by multiple factors like the child’s disease severity and age, as well as the parental support system. Nevertheless, it has been reported that despite the “double burden", caretakers of disabled children who were satisfied with their job also indicated less parenting stress (30). Furthermore, worse mental health was reported in unemployed mothers of chronically ill children. The authors concluded that a lack of childcare services and limited family support increased the likelihood of maternal unemployment [33, 34].

We also found that low social support and limited availability of reliable assistance in the supervision of the CHI child were associated with higher family burden, poor mental health, and decreased PQoL. Social support is important for the adjustment process to a child’s chronic condition. A recent US study found that higher levels of perceived social support were associated with lower levels of anxiety in parents of children with a serious life-threatening illness [35]. In parents of children with cancer, poor social support was the most important predictor of poor mental health outcomes [36].

It is therefore crucial for parents to have a reliable support network and train others in taking care of their children to share the burden of care and improve their well-being.

Parental psychosocial problems can influence both the physical health and psychosocial functioning of the chronically ill child. Nonadherence to treatment and poor disease-related health outcomes in chronically ill children have been linked to their parent’s mental health problems and stress [37–39]. Anxiety or depression in a parent doubles the risk for an adolescent child to also report elevated psychological distress [23]. Interestingly, despite the high psychological distress in parents of CHI children reported here, a Finnish study found no deterioration in the quality of life of the affected children themselves. However, children with comorbidities were excluded from the study [40].

While annual screening for depression and anxiety has been officially recommended, e.g., in patients with cystic fibrosis and their parents [38], these recommendations are lacking for CHI and other chronic diseases. Given the high prevalence of psychosocial distress among parents of chronically ill children and the associated complications, we strongly emphasize the implementation of regular mental health screening of families affected by a child’s chronic illness to identify adverse outcomes early and to optimize referral of parents and/or patients to psychosocial counseling as needed [41].

There are some limitations to this study. First, the disease is rare and heterogeneous; thus, the sample size was relatively small, and parents with psychosocial strains or having children with higher disease severity may be overrepresented in this study due to a higher interest in this matter. However, the high participation rate (48%) contributes to the credibility of the results. Second, because the survey was anonymous, it was not possible to evaluate whether two parents were from the same family. Furthermore, child- and disease-related data were based on parent responses rather than medicals records, and no data were collected on CHI genetics and treatment modalities, which could be additional factors influencing parental mental health. Third, the study cohort presented a relatively homogeneous group with high socioeconomic status, stable relationships, and an overrepresentation of mothers. Future research in larger cohorts across socioeconomic strata is therefore needed to provide adequate information on all parents caring for a child with CHI. Fourth, the comparison of psychosocial burden between parents of children with CHI and those with other chronic diseases is limited by differences in disease characteristics and the use of different screening instruments within studies. Consecutive studies assessing parental psychosocial outcomes in CHI are therefore needed and should favorably use standardized instruments for comparability.

In conclusion, symptoms of anxiety and depression are highly prevalent among parents of children with CHI. Strong predictors of adverse mental health outcomes and lower self-reported PQoL were female gender, sleep disturbance, limited social support, low working hours, and comorbidities or neurological impairment in the affected child. Parents of children with CHI should have annual psychological evaluation using standardized screening instruments, after the child’s initial diagnosis and especially if the affected child has additional comorbidities, to identify psychological problems early and provide psychosocial support as needed. It should be evaluated whether the family’s social support network is sufficient, and parents should be encouraged and supported to train others to take care of their child to share the burden of care and allow more time for personal needs and self-development. Job gratification and distraction from daily coping with the disease through occupational activities may further improve parents’ mental health.

## Data Availability

The data that support the findings of this study are not available publicly but are available from the corresponding author on reasonable request.

## Abbreviations

CGM: Continuous glucose monitoring
CHI: Congenital hyperinsulinism
DSM-IV: Diagnostic and Statistical Manual of Mental Disorders, Fourth Edition
FABEL: Family Burden Questionnaire
GAD-7: Generalized Anxiety Disorder Scale-7
IQR: Interquartile range
*N*: Number
PHQ-8: Patient Health Questionnaire-8
PQoL: Parental quality of life
SD: Standard deviation
ULQIE: Ulm Quality of Life Inventory for Parents of Chronically Ill Children
%: Percent
